# Supplementary material for: Validation of an Eastern Armenian breast cancer health belief survey
Source: PLOS Glob Public Health. 2023 May 5;3(5):e0001849. doi: 10.1371/journal.pgph.0001849 (PMC10162547; doi:10.1371/journal.pgph.0001849)
Supplement: S2 File — (DOCX) [file pgph.0001849.s002.docx]

**ԿՐԾՔԱԳԵՂՁԻ ՔԱՂՑԿԵՂԻ ՍՔՐԻՆԻՆԳԻ ՀԱՐՑԱՇԱՐ**

***Ծանոթություն և ներկայացում***

*[Եթե հեռախոսին պատասխանել Է տղամարդ կամ երեխա] Բարև Ձեզ։ Իմ անունն է ———————, և ես զանգահարել եմ Ձեզ ԵՊԲՀ-ից։ Մենք իրկանացնում ենք ուսումնասիրություն, և ես կցանկանայի զրուցել Ձեր տանը բնակվոց 35-65 տարիքային խմբում գնտվող կնոջ հետ։*

*[Եթե պատասխանում է , որ տանը բնակվում է մեկից ավելի կին նշված տարիքային խմբում, ապա պետք է պատասխանել այսպես]։ Խնդրում եմ փոխանցել հեռախոսը այն կնոջը, ում տարեդարձը վերջերս եք նշել։*

*[Մնացած դեպքերում օգտագործեք հետեևյալ տեքստը* ***Q2.1***

*Իմ անունն է ———————, և ես զանգահարել եմ Ձեզ Երևանի Պետական Բժշկական Համալսարան-ից։ Զանգահարել ենք Ձեզ խնդրանքով, որպեսզի ցանկության դեպքում մասնակցեք մեր հարցազրույցին, որը կատարվում է ԵՊԲՀ-ի և Կալիֆորնիայի համալսարանհի հետ համատեղ ՀՀ առողջապահության նախարարության հովանավորութըամբ։ Մեր նպատակն է հասկանալ, թե հայ կանայք ինչ կարծիք ունեն կրծքագեղձի քաղցկեղի մասին, որոնք են նրանց մտահոգությունները և ինչպես կարող ենք արդյունավետ լուծել դրանք, ինչպես նաև զարգացնել կրծքագեղձի քաղցկեղի արդյունավետ բուժման և սքրինինգային ծրագիր: Հարցազրույցը կտևի մոտ 15 րոպե, կցանկանա՞ք մասնակցություն ունենալ:*

*[եթե մասնակիցը պատասխանում է «ԱՅՈ», շարունակել ըստ ծրագրի, եթե պատասխանում է «ՈՉ» հարցնել ինչու՞մն է հրաժարվելու պատճառը, գրի առնել պատասխանը, շնորհակալություն հայտնել և անջատել: Եթե պատասխանում է, որ հարմար ժամանակ չեք զանգահարել, ճշտել իրեն հարմար ժամը, պայմանավորվել և զանգահարել այդ ժամանակ]:*

**ԲԱՆԱՎՈՐ ՀԱՄԱՁԱՅՆՈՒԹՅՈՒՆ – Q2.2**

*Ձեր հեռախոսահամարը ընտրվել է պատահականության սկզբունքով օգտագործելով պոլիկլինիկայի ռեգիստրը: Մենք չենք պատրաստվում օգտագործել ձեր անձնական տվյալները հետազոտության համար, ինչպիսիք են՝ ձեր անունը, բժշկական պատմությունը և նույնականացնող այլ տեղեկություններ:* *Մասնակցությունը կամավոր է և անանուն, եթե որոշեք մասնակցել չեք ունենա վնաս կամ որևէ նպաստի կորուստ, կարող եք չպատասխանել այն հարցին, որը հարմար չեք գտնում ինչպես նաև ցանկացած պահի կարող եք դադարեցել հարցումը:*

*Եթե հետագայում հարցեր ծագեն, ապա Դուք կարող եք հեռաձայնել նշված հեռախոսահամարով՝ 011 621018։*

*Հետազոտության վերաբերյալ հարցեր ունե՞ք: Կցանկանա՞ք մասնակցել:*

**ՆԵՐԱԾՈՒԹՅՈՒՆ – Q2.3**

*Հետևյալ հարցերը կտրամադրեն անգնահատելի տեղեկատվություն, որը կօգնի Հայաստանում կրծքագեղձի քաղցկեղի հետազոտման ծրագրի մշակմանը: Այնուամենայնիվ, որոշ հարցեր կարող են լինել զգայուն կամ անձնական բնույթի: Կարևոր է, որ դուք գիտեք, որ մասնակցությունը այս հետազոտությանը կամավոր է, և ցանկացած պահի կարող եք փոխել Ձեր կարծիքը հետազոտությանը մասնակցելու վերաբերյալ:*

***ՀԱՄԱՊԱՏԱՍԽԱՆՈՒԹՅԱՆ ՈՐՈՇՈՒՄ***

*Մինչ սկսելը պետք է որոշել ծրագրին Ձեր համապատասխանությունը: -****Q3.1***

1. Քանի՞ տարեկան եք (արդեն լրացած տարիք, թիրախային տարիք 35-65) **Q3.2**
2. Ձեզ մոտ երբևէ ախտորոշվե՞լ է կրծքագեղձի քաղցկեղ **Q3.3**
3. Երբևէ ունեցե՞լ եք կրծքագեղձի վիրահատություն **Q3.4**

*[Եթե մասնակիցը թիրախային տարիքին չի համապատախանում կամ նրա մոտ երբևէ ախտորոշվել է կրծքագեղձի քաղցկեղ կամ ունեցել է վիրահատություն ուռուցքը հեռացնելու համար, ապա չի կարող մասկացել հարցմանը, այդ դեպքում պետք է ասել.]*

*- Հաշվի առնելով Ձեր պատասխանները Դուք չեք համապատասխանում այս հարցաթերթիկի թիրախային խմբին, շնորհակալ ենք Ձեր ժամանակը տրամադրելու և պատրաստակամության համար: Ունե՞ք ինչ-որ հարցեր:* ***Q3.5***

**ԱՆՁՆԱԿԱՆ ՓՈՐՁ/ԳԻՏԵԼԻՔՆԵՐ ԿՐԾՔԱԳԵՂՁԻ ՔԱՂՑԿԵՂԻ ԱԽՏՈՐՈՇՄԱՆ ՀԵՏ**

1. Հավատու՞մ եք արդյոք, որ կրծքագեղձի քաղցկեղը հնարավոր է արդյունավետորեն բուժել, եթե վաղ հայտնաբերվի: **Q4.1**

ա. Այո

բ. Ոչ

գ. Վստահ չեմ

1. Դուք ունե՞ք կրծքագեղձի քաղցկեղի ախտորոշումով մոտ արյունակից ազգական (օրինակ մայր, քույր, մորաքույր, հորքույր, տատ): *[Եթե «Այո», անցնել 4-րդ ապա 5-րդ հարցին, եթե «Ոչ» անցնել 6-րդ հարցին]:* **Q4.2**

*ա.Այո*

բ. Ոչ

1. *[Եթե մասնակիցը պատասխանել է. «Այո» 3-րդ հարցին]* Ո՞ր տարիքում է ախտորոշվել ձեր հարազատի մոտ: **Q4.3**

ա. Մինչև 30

բ. 30-40

գ. 40-60

դ. 60-70

ե. 70-ից հետո

զ. Վստահ չեմ

1. *[Եթե մասնակիցը պատասխանել է. «Այո» 3-րդ հարցին]* Արդյո՞ք կրծքագեղձի քաղցկեղի ախտորոշումը հանգեցրել է ձեր հարազատի մահվան **Q4.4**

ա. Այո

բ. Ոչ

1. Գիտե՞ք ինչ-որ մեկին, ով ձեր արյունակից ազգականը չէ (օրինակ `ընկեր, հարևան, շոու-բիզնեսի ներկայացուցիչ), ում մոտ ախտորոշվել է կրծքագեղձի քաղցկեղ: *[Եթե «Այո», ապա անցեք 7-րդ, ապա 8-րդ հարցին, եթե պատասխանը «Ոչ» է, անցեք 9-րդ հարցին]:* **Q4.5**

ա.Այո

բ. Ոչ

1. Եթե այո, ապա ո՞ր տարիքում է ախտորոշվել: **Q4.6**

ա. Մինչև 30

բ. 30-40

գ. 40-60

դ. 60-70

ե. 70-ից հետո

զ. Վստահ չեմ

1. Եթե պատասխանել եք այո 7-րդ հարցին, արդյո՞ք կրծքագեղձի քաղցկեղի ախտորոշումը հանգեցրել է մահվան: **Q4.7**

ա. Այո

բ. Ոչ

գ. Վստահ չեմ

**ԿՐԾՔԱԳԵՂՁԻ ՔԱՂՑԿԵՂԻ ՍՔՐԻՆԻՆԳԻ ՎԵՐԱԲԵՐՅԱԼ ՏԵՂԵԿԱՑՎԱԾՈՒԹՅՈՒՆ և ՎԵՐԱԲԵՐՄՈՒՆՔ**

1. Լսե՞լ եք հետազոտությունների, ախտորոշումների մասին, որոնք կարող են վաղ հայտնաբերել կրծքագեղձի քաղցկեղը: *[Եթե «Այո» է, անցեք 10-րդ հարցին. Եթե պատասխանը «Ոչ» է, անցնեք 11- րդ հարցին]:* **Q5.1**

ա.Այո

բ. Ոչ

1. Եթե այո, որո՞նք են դրանք: *[նշեք այնքան, որքան մասնակիցը կարող է նշել առանց նշելու տարբերակները]* **Q5.2**

ա.Մամոգրաֆիա
բ. Կրծքագեղձի ուլտրաձայնային հետազոտություն
գ. Կրծքագեղձի մագնիսառեզոնանսային հետազոտություն ՄՌՏ (MRI, ՅԱՄԵՐ)
դ. Կրծքագեղձի կլինիկական զննում բժշկի կողմից
ե. Կրծքագեղձի ինքնազննում

զ. Վստահ չեմ

է. Այլ

1. Երբևէ կատարե՞լ եք կրծքագեղձի ինքնազննում *[Եթե «Այո», անցեք 12-րդ հարցին, եթե պատասխանը «Ոչ» է, անցեք 13- րդ հարցին]:* **Q5.3**

ա.Այո

բ. Ոչ

1. Եթե այո, որքան հաճախ եք դա կատարում: **Q5.4**

ա. Գրեթե ամեն շաբաթ
բ. Ամեն ամիս
գ. Ամեն տարի
դ. Անցած տարիներին մի քանի անգամ կատարվել է

1. Երբևէ անցե՞լ եք կրծքագեղձի քաղցկեղի ճառագայթային սքրինինգ հետազոտություն*: [Եթե «Այո», ապա անցեք 14-րդ հարցի. Եթե պատասխանը «Ոչ» է, անցեք 18-րդ հարցին]:* **Q5.5**

ա.Այո

բ. Ոչ

1. Եթե այդպես է, ո՞րն էր այդ հետազոտությունը: *[Նշեք այնքան, որքան մասնակիցը կարող է նշել]* **Q5.6**

ա.Մամոգրաֆիա
բ. Կրծքագեղձի ուլտրաձայնային հետազոտություն
գ. Կրծքագեղձի մագնիսառեզոնանսային հետազոտություն ՄՌՏ (MRI, ՅԱՄԵՐ)
դ. Կրծքագեղձի կլինիկական զննում բժշկի կողմից
ե. Կրծքագեղձի ինքնազննում

զ. Վստահ չեմ

է. Այլ չնշված

1. Ե՞րբ եք վերջին անգամ ճառագայթաբանական հետազոտություն իրականացրել կրծքագեղձի քաղցկեղի սքրինինգի համար: *[Եթե պատասխանել է «զ – երբեք», ապա անցեք հարց 17-ին]* **Q5.7**

ա. Ավելի քիչ, քան մեկ տարի առաջ
բ. Անցյալ տարի
գ. 2-5 տարի առաջ
դ. 5-10 տարի առաջ
ե. Ավելի քան 10 տարի առաջ
զ. Երբեք

1. Ինչո՞ւ մասնակցեցիք կրծքագեղձի քաղցկեղի սքրինինգին: **Q5.8**

ա. Իմ բժիշկը խորհուրդ տվեց և ուղեգրեց ինձ կրծքագեղձի սքրինինգ հետազոտության
բ. Ես խնդրեցի իմ բժշկին, որ ինձ ուղեգրի կրծքագեղձի սքրինինգ հետազոտության

գ. Սքրինինգ եմ իրականացրել առանց իմ բժշկի պաշտոնական ուղեգրման
դ. Ընտանիքի անդամը կամ ընկերը խորհուրդ են տվել սքրինինգ անցնել

ե. Այլ պատճառներով

1. Դուք կանցնե՞ք կրծքագեղձի ճառագայթաբանական սքրինինգ հետազոտություն **Q5.9**

ա.Այո

բ. Ոչ

գ. Վստահ չեմ

1. Ո՞ր հետազոտությունն եք նախընտրում կրծքագեղձի քաղցկեղի սքրինինգի համար։ **Q5.10**

ա.Մամոգրաֆիա
բ. Կրծքագեղձի ուլտրաձայնային հետազոտություն
գ. Կրծքագեղձի մագնիսառեզոնանսային հետազոտություն ՄՌՏ (MRI, ՅԱՄԵՐ)
դ. Կրծքագեղձի կլինիկական զննում բժշկի կողմից
ե. Կրծքագեղձի ինքնազննում

1. Խոսե՞լ եք Ձեր բժշկի հետ կրծքագեղձի քաղցկեղի կամ դրա սքրինինգի մասին: **Q5.11**

ա.Այո

բ. Ոչ

1. Ձեր բժիշկը Ձեզ հետ քննարկե՞լ կամ առաջարկե՞լ է կրծքագեղձի քաղցկեղի սքրինինգ: **Q5.12**

ա.Այո

բ. Ոչ

1. Որքա՞ն հավանական է, որ դուք անցնեք սկրինիգային մամոգրաֆիա կամ ուլտրաձայնային հետազոտություն, եթե ձեր բժիշկը խորհուրդ է տալիս: **Q5.13**

ա. Շատ հավանական է
բ. Հավանական է
գ. Համոզված չեմ/չգիտեմ

դ. Քիչ հավանական է

ե. Անհնարին է

**INTRODUCTION TO MODIFIED CHBMS**

*Մենք ուզում ենք ուսումնասիրել ձեր կարծիքները, զգացողությունները և տեղեկացվածությունը կրծքագեղձի քաղցկեղի և դրա հետազոտության/սքրինինգի վերաբերյալ: Խնդրում եմ, փորձեք պատասխանել որքան հնարավոր է անկեղծ: Բոլոր հարցերը լինելու են 1-5 սանդղակով: Հարցերի առաջին խմբի համար դուք կգնահատեք տրված իրավիճակի հավանականությունը:* **Q6.1**

*«Կրծքագեղձի ճառագայթաբանական սքրինինգ հետազոտություն» տերմինը, որը դուք բազմիցս կլսեք այս հարցման ընթացքում, վերաբերում է առկա ռադիոգրաֆիկ հետազոտություններին, որոնք օգտագործվում են կրծքագեղձի քաղցկեղի սքրինինգի համար, որը ներառում է մամոգրաֆիա, կրծքագեղձի ուլտրաձայնային հետազոտություն, մագնիսառեզոնանսային հետազոտություն ՄՌՏ/MRI: Այս պահից սկսած «Կրծքագեղձի ճառագայթաբանական սքրինինգ հետազոտություն» տերմինը, որի սահամանումը ես քիչ առաջ Ձեզ ներկայացրեցի, կփոխարինվի ավելի կարճ «սքրինինգ հետազոտություն» անվամբ.* **Q6.2**

*Քիչ անց հնչող հարցերոմ Դուք գնահատելու եք ներկայացված իրավիճակի հավանականությունը 1-5 սանդղակով, որտեղ «1» գնահատումը նշանակում է անհնար, «2» -ը նշանակում է, որ շատ անհավանական է, «3» -ը նշանակում է, որ վստահ չեք, «4» -ը նշանակում հավանական է, իսկ «5» -ը ՝ շատ հավանական: Մինչ սկսելը ունե՞ք հարցեր կամ պարզաբանումների անհրաժեշտություն։* **Q6.3**

| Q6.3 | Մտավախություն (1=անհնար է, 5=շատ հավանական է) | 1 | 2 | 3 | 4 | 5 |
| --- | --- | --- | --- | --- | --- | --- |
| 1 | Որքա՞ն հավանական է այն, որ 5 տարի հետո ես կրծքագեղձի քաղցկեղ ձեռք կբերեմ: |  |  |  |  |  |
| 2 | Որքա՞ն հավանական է այն, որ 10 տարի հետո ես կրծքագեղձի քաղցկեղ ձեռք կբերեմ : |  |  |  |  |  |
| 3 | Որքա՞ն հավանական է այն, որ իմ կյանքի ընթացքում կրծքագեղձի քաղցկեղ ձեռք կբերեմ : |  |  |  |  |  |
| 4 | Իմ տարիքի մյուս կանանց համեմատ, որքա՞ն հավանական է, որ ինձ մոտ կախտորոշեն կրծքագեղձի քաղցկեղ: |  |  |  |  |  |
|  |  |  |  |  |  |  |
| Q6.4 | **Օգուտներ (1=անհնար է, 5=շատ հավանական է)** | 1 | 2 | 3 | 4 | 5 |
| 5 | Եթե կրծքագեղձի քաղցկեղը հայտնաբերվի վաղ, որքանո՞վ է հավանական այն, որ քաղցկեղը հաջողությամբ կբուժվի: |  |  |  |  |  |
| 6 | Որքա՞ն հավանական է այն, որ կրծքագեղձի մամոգրաֆիան կօգնի ինձ հայտնաբերել կրծքագեղձի քաղցկեղը, երբ այն հենց նոր է սկսում: |  |  |  |  |  |
| 7 | Որքա՞ն հավանական է այն, որ կրծքագեղձի մամոգրաֆիան կօգնի ինձ հայտնաբերել կրծքագեղձի քաղցկեղը, նախքան դրա բավականաչափ մեծանալը զգալու համար: |  |  |  |  |  |
| 8 | Որքա՞ն հավանական է այն, որ կրծքագեղձի մամոգրաֆիան կնվազեցնի կրծքագեղձի քաղցկեղից մահանալու իմ հավանականությունը: |  |  |  |  |  |

*Հաջորդ հարցերի համար կփորձեք գնահատել, թե որքանով եք համաձայն կամ համաձայն չեք տվյալ հարցի կամ իրավիճակի հետ: Երբ պատասխանեք «1», դուք ընդհանրապես համաձայն չեք այդ հարցապնդման հետ, «2»-ը ինչ-որ չափով համաձայն չեք, «3» -ը ՝ վստահ չեք, «4»-ը ինչ-որ չափով համաձայն եք, «5» -ը հիմնականում կամ լիովին համաձայն եք: Մինչ սկսելը ունե՞ք հարցեր կամ պարզաբանումների անհրաժեշտություն (****Q7.1)***

| Q7.1 | Խոչընդոտներ (1 = ընդհանրապես համաձայն չեմ, 5 = լիովին համաձայն եմ) | 1 | 2 | 3 | 4 | 5 |
| --- | --- | --- | --- | --- | --- | --- |
| 9 | Ինձ համար անհարմար կլիներ կրծքագեղձի մամոգրաֆիան : |  |  |  |  |  |
| 10 | Մամոգրաֆիան կարող է առաջացնել կրծքագեղձի քաղցկեղ: |  |  |  |  |  |
| 11 | Բուժումը, որը ես կստանամ կրծքի քաղցկեղի դեմ, ավելի վատ կլինի, քան ինքնին քաղցկեղը: |  |  |  |  |  |
| 12 | Կրծքագեղձի քաղցկեղի դեմ բուժվելը ինձ համար շատ խնդիրներ կառաջացնի |  |  |  |  |  |
| 13 | Առողջական այլ խնդիրները ինձ հետ կպահեն մամոգրաֆիա իրականացնելուց : |  |  |  |  |  |
| 14 | Իմ տարիքը ինձ հետ կպահի մամոգրաֆիա իրականացնելուց: |  |  |  |  |  |
| 15 | Ես չեմ անցնի մամոգրաֆիա կամ կրծքագեղձի այլ ճառագայթային հետազոտություն, քանի-որ բժիշկս արդեն զննել է իմ կրծքերը |  |  |  |  |  |
| 16 | Կրծքագեղձի ուռուցք հայտնաբերլու վախը ինձ հետ կպահի մամոգրաֆիա անցնելուց: |  |  |  |  |  |
| 17 | Մամոգրաֆիա անցնելու դժվարությունը ինձ հետ կպահի այն անցնելուց |  |  |  |  |  |
| 18 | Մամոգրաֆիայի ընթացքում ցավի մասին մտահոգությունը ինձ հետ կպահի հետազոտություն անցնելուց |  |  |  |  |  |
| 19 | Ամոթի զգացումը կապված մերկ կրծքի հետազոտման հետ, ինձ հետ կպահի մամոգրաֆիա իրականացնելուց |  |  |  |  |  |
| 20 | Ես ժամանակ չունեմ կրծքագեղձի մամոգրաֆիա անցնելու համար |  |  |  |  |  |
| 21 | Կրծքագեղձի սքրինինգ հետազոտության համար գումար չունենալը ինձ հետ կպահի հետազոտություն անցնելուց |  |  |  |  |  |
| 22 | Կրծքագեղձի քաղցկեղի մասին անհանգստությունը ինձ հետ կպահի իրականացնել մամոգրաֆիա: |  |  |  |  |  |
| 23 | Ռենտգենյան ճառագայթահարման հետ կապված մտահոգությունները ինձ հետ կպահեն մամոգրաֆիա իրականացնելուց: |  |  |  |  |  |
| 24 | Ես գտնում եմ, որ դժվար է հիշել՝ մամոգրաֆիա իրականացնելու համար բժշկի հետ ժամադրության օրն ու ժամը։ |  |  |  |  |  |
| 25 | Բժշկի հետ ժամադրության մասին մոռանալու ռիսկը ինձ հետ կպահի մամոգրաֆիա իրականացնելուց |  |  |  |  |  |
| 26 | Կրծքագեղձի հետազոտման կենտրոններում կոպիտ վերաբերմունքը ինձ հետ կպահի մամոգրաֆիա իրականացնելուց |  |  |  |  |  |
| 27 | Քաղցկեղի մասին չտեղեկանալու ցանկությունը ինձ հետ կպահի մամոգրաֆիա իրականացնելուց |  |  |  |  |  |
| 28 | Չեմ ցանկանում կրծքագեղձի սքրինինգ հետազոտություն անցնել, քանի որ եթե քաղցկեղը հայտնաբերվի, վախենում եմ, որ չեմ կարողանա վճարել իմ բուժման համար |  |  |  |  |  |
| 29 | Չեմ ցանկանում մամոգրաֆիա անցնել, քանի-որ չեմ վստահում իմ բժիշկներին և նրանց հմտություններին |  |  |  |  |  |
| 30 | Չեմ ցանկանում մամոգրաֆիա անցնել, քանի-որ քաղցկեղը տրված է Աստծո կողմից և սքրինինգը միևնույն է դրա բուժումը հնարավոր չի դարձնի |  |  |  |  |  |
| 31 | Չեմ ցանկանում մամոգրաֆիա անցնել, քանի-որ եթե հայտնաբերվի քաղցկեղ վախենում եմ որ կկորցնեմ կրծքերս |  |  |  |  |  |
| 32 | Չեմ ցանկանում կրծքագեղձի սքրինինգ հետազոտություն անցնել, քանի որ վախենում եմ խոսել կրծքագեղձի քաղցկեղի մասին |  |  |  |  |  |
|  | *Հիշեցնեմ, երբ պատասխանեք «1», դուք ընդհանրապես համաձայն չեք այդ հարցապնդման հետ, «2»-ը ինչ-որ չափով համաձայն չեք, «3» -ը ՝ վստահ չեք, «4»-ը ինչ-որ չափով համաձայն եք, «5» -ը հիմնականում կամ լիովին համաձայն եք: Մինչ սկսելը ունե՞ք հարցեր կամ պարզաբանումների անհրաժեշտություն* |  |  |  |  |  |
| Q7.2 | **Ինքնարդյունավետություն (1=ընդհանրապես համաձայն չեմ, 5=լիովին համաձայն եմ)** | 1 | 2 | 3 | 4 | 5 |
| 33 | Ես կարող եմ մամոգրաֆիա անցնել, նույնիսկ եթե բժիշկս չի ասում, որ անցնեմ այն |  |  |  |  |  |
| 34 | Ես կարող եմ գտնել փոխադրամիջոց կրծքագեղձի սքրինինգ հետազոտություն անցնելու համար |  |  |  |  |  |
| 35 | Ես կարող եմ օրվա ընթացքում այլ բաներ այնպես կազմակերպել, որպեսզի մամոգրաֆիա անցնեմ |  |  |  |  |  |
| 36 | Ես կարող եմ խոսել կրծքագեղձի սքրինինգ հետազոտության կենտրոնում գտնվող մարդկանց հետ, եթե խնդիր ունենամ |  |  |  |  |  |
| 37 | Ես կանցնեմ մամոգրաֆիա, նույնիսկ եթե անհանգստանում եմ քաղցկեղի առկայության մտքից։ |  |  |  |  |  |
| 38 | Ես կանցնեմ մամոգրաֆիա , նույնիսկ եթե չգիտեմ, թե ինչ արդյունքի սպասել |  |  |  |  |  |
| 45 | Ես կարող եմ ինքնուրույն գրանցվել կրծքագեղձի սկրինիգային հետազոտության համար |  |  |  |  |  |
| 46 | Ես կարող եմ գտնել հարմար բուժ-հաստատություն, մամոգրաֆիա իրականացնելու համար |  |  |  |  |  |
| 47 | Շատ հավանական է որ կանցնեմ մամոգրաֆիկ հետազոտություն |  |  |  |  |  |
|  |  |  |  |  |  |  |
| Q7.3 | **Վախ (1=ընդհանրապես համաձայն չեմ,5=լիովին համաձայն եմ)** | 1 | 2 | 3 | 4 | 5 |
| 48 | Երբ մտածում եմ կրծքագեղձի քաղցկեղի մասին, վախենում եմ |  |  |  |  |  |
| 49 | Երբ մտածում եմ կրծքագեղձի քաղցկեղի մասին, նյարդայնանում եմ |  |  |  |  |  |
| 50 | Երբ մտածում եմ կրծքագեղձի քաղցկեղի մասին,վրդովվում եմ նեղվում եմ |  |  |  |  |  |
| 51 | Երբ մտածում եմ կրծքագեղձի քաղցկեղի մասին, ընկճվում եմ |  |  |  |  |  |
| 52 | Երբ մտածում եմ կրծքագեղձի քաղցկեղի մասին, սիրտս արագ է աշխատում |  |  |  |  |  |
| 53 | Երբ մտածում եմ կրծքագեղձի քաղցկեղի մասին, անհանգստանում եմ |  |  |  |  |  |

**Վճարելու պատրաստակամություն**

54*. [Ազատ պատասխան/պատասխան ըստ ցանկության]* ՀՀ դրամով որքան եք պատրաստ վճարել կրծքագեղձի քաղցկեղի հետազոտման համար, եթե Ձեր բժիշկը խորհուրդ է տալիս իրականացնել այն: *[Եթե միջակայք է նախատեսված, խնդրում ենք վերցրեք ստորին սահմանը]* **Q8.1**

55. *[Ազատ պատասխան]* ՀՀ դրամով որքան եք պատրաստ վճարել կրծքագեղձի քաղցկեղի հետազոտման համար, եթե կան ազգային ուղեցույցներ: *[Եթե միջակայք է նախատեսված, խնդրում ենք վերցրեք ստորին սահմանը]* **Q8.2**

56. Դուք ունե՞ք սոցիալական փաթեթ կամ առողջության ապահովագրության: **Q8.3**

ա. Այո

բ. Ոչ

**ժողովրդագրական հարցեր**

*Հետազոտության վերջին բաժինը ներառում է հակիրճ ժողովրդագրական հարցեր, ինչպիսիք են ընտանեկան կարգավիճակը, զբաղվածությունը և ամսական ծախսերը: Ես ձեզ կտրամադրեմ պատասխանի ընտրություններ.* **Q9.1**

57. Ո՞րն է ձեր ամուսնական կարգավիճակը: **Q9.2**

ա. միայնակ

բ. ամուսնացած

գ. Բաժանված

դ. Այրի

58. Ո՞րն է ձեր ամենաբարձր կրթական մակարդակը **Q9.3**

ա. Թերի միջնակարգ

բ. Միջնակարգ

գ. Միջին մասնագիտական

դ. Բարձրագույն

59. Ո՞րն է աշխատանքային ներկայիս կարգավիճակը: **Q9.4**

ա. Աշխատում եմ ամբողջ դրույքով

բ. Աշխատում եմ կես դրույքով

գ. Գործազուրկ

դ. Այլ

60. Որքա՞ն են ձեր ընտանիքի ամսական ծախսերը: **Q9.5**

ա. 100 000 դրամից ցածր

բ. 100,000 - 300,000 դրամ

գ. 300,000 - 500,000 դրամ

դ. 500 000 դրամից բարձր

61. Ո՞ր հարցապնդումն է ձեզ ամենալավը նկարագրում: **Q9.6**

ա. Ես աթեիստ եմ

բ. Ես շատ հավատացյալ չեմ և հազվադեպ եմ հաճախում եկեղեցի կամ այլ կրոնական հավաքույթներ

գ. Ես հավատացյալ եմ, բայց կանոնավոր չեմ հաճախում եկեղեցի կամ այլ կրոնական հավաքույթներ

դ. Ես հավատացյալ մարդ եմ և պարբերաբար հաճախում եմ եկեղեցի կամ այլ կրոնական հավաքույթներ

62. Ինչպե՞ս կբնութագրեք ձեր առողջությունը: **Q9.7**

ա Լավ

բ. Բավարար

գ. Անբավարար

63. Երևանյան ո՞ր վարչական շրջանում եք տվյալ պահին բնակվում։ **Q9.9**

ա.Կենտրոն
բ. Նորք-Մարաշ
գ. Ավան
դ. Նոր Նորք
ե. Քանաքեռ/Զեյթուն

զ. Արաբկիր

է. Աջափնյակ

ը. Շենգավիթ

թ. Էրեբունի

ժ. Մալաթիա/Սեբաստիա

ի. Դավթաշեն

լ. Նուբարաշեն

*Սա եզրափակում է մեր հարցումը: Շատ շնորհակալ եմ ձեր մասնակցության համար: Ձեր ներդրումը էական նշանակություն ունի Հայաստանում կրծքագեղձի քաղցկեղի արդյունավետ կանխարգելման համար: Դուք ընտրվել եք, որպեսզի կրկին մասնակցեք հարցմանը մոտ 2 շաբաթվա ընթացքում: Դուք համաձա՞յն եք որպեսզի Ձեզ կրկին զանգահարեն (եթե պատասխանը«Այո» է շնորհակալություն հայտնեք նրանց և հարցրեք. «Ո՞րը կլինի լավագույն օրն ու ժամը 10-14 օրվա ընթացքում զանգահարելու համար»: Եթե պատասխանը «Ոչ» է, շնորհակալություն հայտնեք նրանց): Որևէ՞ հարց կամ մտահոգություն, մինչև այսօր ավարտելը:* **Q9.14**
