# Supplementary material for: Validation of an Eastern Armenian breast cancer health belief survey
Source: PLOS Glob Public Health. 2023 May 5;3(5):e0001849. doi: 10.1371/journal.pgph.0001849 (PMC10162547; doi:10.1371/journal.pgph.0001849)
Supplement: S3 File — (DOCX) [file pgph.0001849.s003.docx]

**BREAST CANCER SCREENING QUESTIONNAIRE –– US VALIDATION**

***Self Introduction***

*[In remaining situations, use the following text].* **Q2.1**

My name is ________________, and I am calling you from UCLA. We are calling to kindly ask for your participation in a questionnaire, which is jointly conducted by YSMU and UCLA under sponsorship of Ministry of Health of Armenia. Our aim is to understand how Armenian women feel about breast cancer, what their concerns are, and how we can address those concerns effectively. Our main goal is to develop an effective breast cancer screening and treatment in Armenia. The questionnaire will take about 15 minutes. Are you willing to participate?

[*If the participant replies “Yes” proceed with the consent; if ‘No’ ask “What is the reason for not participating?” document the reason, thank the participant and hang up; if the reply is “This is not a good time,” ask for a better time to call and arrange for a later phone call at a more convenient time for the*

**VERBAL CONSENT Q2.2**

Your phone number was obtained in a randomized fashion by using the registry at your polyclinic. The registry does not contain any information pertaining to your medical records, and only includes your name, age, gender, and contact information including registered address and phone number. We are not going to use any of your personal information for the study, such as your name, medical history, and other identifying information. There is no risk of a breach of confidentiality. I will not link your name to anything you say within the survey. Participation is voluntary. If you decide not to participate, there will be no penalty or loss of benefits, to which you are otherwise entitled. You can, of course, decline to answer any questions, as well as stop participating at any time, without any penalty or loss of benefits, to which you are otherwise entitled. If questions arise in the future, feel free to contact us by 8184252233. Do you have questions regarding our study? Would you like to proceed?

**DISCLAIMER AND INTRODUCTION TO THE QUESTIONNAIRE Q2.3**

The following questions will provide invaluable information that will help with developing a breast cancer screening program in Armenia; however, some questions may be sensitive or personal in nature. It is important that you know participation in this survey is voluntary and you can change your mind regarding participation at any time.

Before we start, we will need to determine your eligibility. **Q3.1**

**SCREEN FOR INCLUSION/EXCLUSION CRITERIA**

1. What is your age? (target age range 35-65) **Q3.2**
2. Have you ever been diagnosed with breast cancer? **Q3.3**
3. Have you ever had breast surgery to remove a tumor? **Q3.4**

*[if the participant is not within the target age range, has had breast cancer or breast surgery for a known or unknown tumor, they are not eligible for the study; if that is the case here is the script*

- Given your answers, you are not eligible for this questionnaire. Thank you very much for your time and willingness to participate. Do you have any questions or concerns?

*[if the inclusion criteria have been met, and exclusion criteria effectively ruled out, then smoothly proceed with question number 1]* **Q3.5**

**EXPOSURE TO BREAST CANCER**

1. Do you believe that breast cancer can be effectively treated if caught early? **Q4.1**
   1. Yes
   2. No
   3. Unsure
2. Have you had a first or second-degree blood relative (mother, sister, aunt, grandmother) diagnosed with breast cancer? [*If “Yes”, proceed to question 4 then 5, If the answer is “No”, proceed to question 6].* **Q4.2**
   1. Yes
   2. No
3. [*If participant answered “Yes to question 3]* In which age range was the diagnosis reported in your relative? **Q4.3**
   1. Less than 30
   2. 30-40
   3. 40-60
   4. 60-70
   5. Above 70
4. [*If participant answered “Yes to question 3]* Did the diagnosis of breast cancer result in the mortality of your relative? **Q4.4**
   1. Yes
   2. No
5. Do you know someone, who is not a blood relative of yours (e.g. friend, neighbor, showbusiness personality) diagnosed with breast cancer? [*If “Yes”, proceed to question 7 then 8, If the answer is “No”, proceed to question 9].* **Q4.5**
   1. Yes
   2. No
6. If Yes, in which age range was she diagnosed? **Q4.6**
   1. Less than 30
   2. 30-40
   3. 40-60
   4. 60-70
   5. Above 70
   6. Unsure
7. If you answered yes to question 7, did the diagnosis of breast cancer result in mortality? **Q4.7**
   1. Yes
   2. No
   3. Unsure

**BREAST CANCER SCREENING AWARENESS AND BEHAVIOR**

1. Have you heard of exams, diagnostics that could detect breast cancer early? [*If “Yes”, proceed to question 10, If the answer is “No”, proceed to the question 11].* **Q5.1**
   1. Yes
   2. No
2. If Yes, what are they? [*check as many as the participant can name]* **Q5.2**
   1. Mammography
   2. Breast ultrasound
   3. Breast MRI
   4. Breast clinical exam by physician
   5. Breast self-examination
   6. Unsure
   7. Other
3. Have you ever performed a breast self-examination? [*If “Yes”, proceed to question 12, If the answer is “No”, proceed to the question 13]* **Q5.3**
   1. Yes
   2. No
4. If yes how often do you perform it? **Q5.4**
   1. Almost Weekly
   2. Monthly
   3. Yearly
   4. Performed few times in the past years
5. Have you ever had breast cancer screening performed? [*If “Yes”, proceed to question 14. If the answer is “No”, proceed to question 18].* **Q5.5**
   1. Yes
   2. No
6. If so, what was the exam? [*check as many as the participant can name]* **Q5.6**
   1. Mammography
   2. Breast ultrasound
   3. Breast MRI
   4. Breast clinical exam by physician
   5. Unsure
   6. Other test (not listed)
7. When was the last time you had imaging performed for breast cancer screening? **Q5.7**
   1. Less than a year ago
   2. Past year
   3. 2-5 years ago
   4. 5-10 years ago
   5. More than 10 years ago
   6. Never
8. Why did you participate in breast cancer screening? **Q5.8**
   1. My doctor strongly recommended, and referred me to breast imaging
   2. I asked my doctor to be referred for breast imaging
   3. I obtained screening without the formal recommendation of my physician
   4. A family member or friend recommended obtaining screening
   5. For other unlisted reasons
9. Would you consider obtaining breast imaging for screening? **Q5.9**
   1. Yes
   2. No
   3. Unsure
10. Which screening modality to you prefer? **Q5.10**
    1. Mammography
    2. Breast ultrasound
    3. Breast MRI
    4. Breast clinical exam by physician
    5. Breast self-examination
11. Have you talked to your doctor about breast cancer and/or its screening? **Q5.11**
    1. Yes
    2. No
12. Has your doctor discussed or recommended breast cancer screening to you? **Q5.12**
    1. Yes
    2. No
13. How likely are you to do a screening mammogram or ultrasound if your doctor recommends one? **Q5.13**
    1. Very likely
    2. Likely
    3. Unsure
    4. Unlikely
    5. Impossible

**INTRODUCTION TO MODIFIED CHBMS**

We want to explore your opinions, feelings and beliefs about breast cancer and its screening. Please, try to answer as honestly as you can. All questions are going to be in 1-5 scale. For the first set of questions you are going to rate the likelihood of a given situation. **Q6.1**

The term ‘breast imaging’ that you will hear multiple times throughout the survey refers to established radiographic modalities that are used for breast cancer screening, which include mammography, breast ultrasound, breast MRI. **Q6.2**

The following questions will need to be rated based on 1-5 scale, where rating of ‘1’ means impossible, ‘2’ means highly unlikely, ‘3’ means that you are unsure, ‘4’ means likely, and ‘5’ means very likely. Any questions or clarifications before we start? [*After proper clarification, you may pay proceed with the questions].* **Q6.3**

| Q6.3 | Susceptibility (1=impossible, 5=extremely likely) | 1 | 2 | 3 | 4 | 5 |
| --- | --- | --- | --- | --- | --- | --- |
| 1 | How likely is that I will get breast cancer in 5 years? |  |  |  |  |  |
| 2 | How likely is that I will get breast cancer in 10 years? |  |  |  |  |  |
| 3 | How likely is that I will get breast cancer in my lifetime? |  |  |  |  |  |
| 4 | Compared to other women of my age, how likely am I to be diagnosed with breast cancer? |  |  |  |  |  |
|  |  |  |  |  |  |  |
| Q6.4 | **Benefits (1=impossible, 5=extremely likely)** | 1 | 2 | 3 | 4 | 5 |
| 5 | If breast cancer was found early, how likely is it that the cancer could be successfully treated? |  |  |  |  |  |
| 6 | How likely is that having a breast imaging would help me find breast cancer when it is just getting started? |  |  |  |  |  |
| 7 | How likely is that having a breast imaging would help me find breast cancer before it is big enough to feel? |  |  |  |  |  |
| 8 | How likely is that having a breast imaging would decrease my chances of dying from breast cancer? |  |  |  |  |  |

For the next set of questions will try to gauge how much you agree or disagree with a given question or situation. When you answer ‘1’ you complete disagree with the statement, ‘2’ somewhat disagree, ‘3’ unsure, ‘4’ somewhat agree, ‘5’ mostly agree. Any questions or clarifications before we proceed?

| Q7.1 | Barriers (1=strongly disagree, 5=strongly agree) | 1 | 2 | 3 | 4 | 5 |
| --- | --- | --- | --- | --- | --- | --- |
| 9 | Getting breast imaging would be inconvenient for me |  |  |  |  |  |
| 10 | Getting a mammogram or other breast imaging could cause breast cancer |  |  |  |  |  |
| 11 | The treatment I would get for breast cancer would be worse than the cancer itself |  |  |  |  |  |
| 12 | Being treated for breast cancer would cause me a lot of problems |  |  |  |  |  |
| 13 | Other health problems would keep me from having a breast imaging |  |  |  |  |  |
| 14 | My age would keep me from having a breast imaging |  |  |  |  |  |
| 15 | I would not get a mammogram or other breast imaging because my doctor already examines my breasts |  |  |  |  |  |
| 16 | Being afraid of finding a breast tumor would keep me from having a breast imaging |  |  |  |  |  |
| 17 | The trouble of having a mammogram would keep me from getting one |  |  |  |  |  |
| 18 | Concern about pain during a mammogram would keep me from having one |  |  |  |  |  |
| 19 | Being embarrassed about my body would keep me from having breast imaging |  |  |  |  |  |
| 20 | I do not have time to have breast imaging |  |  |  |  |  |
| 21 | Not being able to afford breast imaging would keep me from having one |  |  |  |  |  |
| 22 | Worrying about breast cancer would keep me from having breast imaging |  |  |  |  |  |
| 23 | Concerns about being exposed to the x-ray would keep me from having breast imaging |  |  |  |  |  |
| 24 | I find it difficult to remember to make an appointment for breast imaging |  |  |  |  |  |
| 25 | Forgetting my appointment would keep me from getting breast imaging |  |  |  |  |  |
| 26 | Being treated rudely at the breast imaging centers would keep me from having breast imaging |  |  |  |  |  |
| 27 | Not wanting to know would keep me from having a breast imaging |  |  |  |  |  |
| 28 | I am reluctant to undergo breast imaging for screening, because if cancer is detected I am afraid I will not be able to pay for my treatment |  |  |  |  |  |
| 29 | I am reluctant to undergo breast imaging for screening, because I do not trust my doctors and their skills |  |  |  |  |  |
| 30 | I am reluctant to undergo breast imaging for screening, because breast cancer is given by God, and screening will not make its treatment possible |  |  |  |  |  |
| 31 | I am reluctant to undergo breast imaging for screening, because if cancer is detected, I am afraid I will lose my breasts |  |  |  |  |  |
| 32 | I am reluctant to undergo breast imaging for screening, because I am afraid to talk about breast cancer |  |  |  |  |  |
|  |  |  |  |  |  |  |
| Q7.2 | **Self-Efficacy (1=strongly disagree, 5=strongly agree)** | 1 | 2 | 3 | 4 | 5 |
| 33 | I can get breast imaging for screening even if my doctor does not tell me to get one |  |  |  |  |  |
| 34 | I can get transportation to have breast imaging completed |  |  |  |  |  |
| 35 | I can arrange other things in my day to have breast imaging |  |  |  |  |  |
| 36 | I can talk to people at the breast imaging center if I have a problem |  |  |  |  |  |
| 37 | I will get breast imaging even if I am worried |  |  |  |  |  |
| 38 | I will get breast imaging even if I don’t know what to expect |  |  |  |  |  |
| 45 | I can make an appointment for breast imaging for screening |  |  |  |  |  |
| 46 | I can find a place to have my breast imaging done |  |  |  |  |  |
| 47 | It is very likely that I will get breast imaging for screening. |  |  |  |  |  |
|  |  |  |  |  |  |  |
| Q7.3 | **Fear (1=strongly disagree, 5=strongly agree)** | 1 | 2 | 3 | 4 | 5 |
| 48 | When I think of breast cancer, I get scared |  |  |  |  |  |
| 49 | When I think of breast cancer, I feel nervous |  |  |  |  |  |
| 50 | When I think of breast cancer, I get upset |  |  |  |  |  |
| 51 | When I think of breast cancer, I get depressed |  |  |  |  |  |
| 52 | When I think of breast cancer, my heart beats faster |  |  |  |  |  |
| 53 | When I think of breast cancer, I feel anxious |  |  |  |  |  |

The final section of a survey includes brief questions regarding your demographics such as marital status, employment, and monthly expenditures. I will provide you with answer choices.

**WILLINGNESS TO PAY**

1. Do you have a social package or health insurance plan? **Q8.3**
   a. Yes
   b. No

**DEMOGRAPHIC QUESTIONS**

The last portion of our study involves a brief demographic questionnaire. **Q9.1**

1. What is your marital status? **Q9.2**
   1. Single
   2. Married
   3. Divorced/Separated
   4. Widowed
2. What is the highest educational level that you have achieved? **Q9.3**
   1. Incomplete secondary
   2. Complete secondary
   3. Vocational
   4. Higher education
3. What is your current employment status? **Q9.4**
   1. Employed full-time
   2. Employed part-time
   3. Unemployed
   4. Other
4. Which statement describes you the best? **Q9.6**
   1. I am an atheist
   2. I am not very spiritual, and seldomly attend church or other religious gatherings
   3. I am spiritual, but do not regularly attend church or other religious gatherings
   4. I am spiritual, and regularly attend church or other religious gatherings
5. How would you describe your health? **Q9.7**
   1. Good
   2. Fair
   3. Bad

This concludes our survey. Thank you very much for your participation. Your contribution is essential for effective prevention of breast cancer in Armenia. You have been selected to be re-interviewed in about 2 weeks. Do you agree to be called again? [*if the answer is “Yes,” thank them and ask “When would be the best day and time to call in 10-14 days?” If the answer is “No,” thank them.* Any questions or concerns before we finish today? **Q9.14**
