# Supplementary material for: Validation of an Eastern Armenian breast cancer health belief survey
Source: PLOS Glob Public Health. 2023 May 5;3(5):e0001849. doi: 10.1371/journal.pgph.0001849 (PMC10162547; doi:10.1371/journal.pgph.0001849)
Supplement: Attachment — Submitted filename: Response to Reviewers.docx [file pgph.0001849.s004.docx]

**Response to Reviewers**

Dear Editors,

Thank you for taking the time to review our paper and provide feedback. To simplify, we have separated our responses below by editor. Our comments are in red.

**General Comments:**

- Please review your reference list to ensure that it is complete and correct. If you have cited papers that have been retracted, please include the rationale for doing so in the manuscript text, or remove these references and replace them with relevant current references. Any changes to the reference list should be mentioned in the rebuttal letter that accompanies your revised manuscript. If you need to cite a retracted article, indicate the article’s retracted status in the References list and also include a citation and full reference for the retraction notice. To the best of our knowledge, none of these papers have been retracted. We reached out to the editor via PLOS find out if there were any references that were showing up as retracted but did not receive a response. Citation 14 has been revised to fix a misspelling. Citations 23 (Anthoine et al, 2014) and 25 (Kazungu et al, 2022) have been added to this version. Citations 7-21 are simply examples of the CHBMS that has been translated and adapted to various cultures and contexts across the globe. If one of these examples (7-21) is showing up as retracted, it would not be an issue to remove this citation.
- Please provide additional details regarding participant consent. In the ethics statement in the Methods and online submission information, please ensure that you have specified what type you obtained (for instance, written or verbal, and if verbal, how it was documented and witnessed). If your study included minors, state whether you obtained consent from parents or guardians. If the need for consent was waived by the ethics committee, please include this information. We have specified that consent was obtained verbally and was recorded by trained local surveyors. Given the age range of participants (35-65), no minors were included. Please see lines 95-97.
- Please amend your Data Availability Statement and indicate where the data may be found. We have included our de-identified data as a supplement “S1.”
- Additional Notes from Authors: When preparing our data to be included as a supplementary file, we noticed that one respondent missing ~ 1/3 of the CHBMS responses was initially included in the analysis that should have been removed as incomplete. The data has been re-analyzed to account for removal of this one respondent (resulting in very slight changes to tables 1 and 4). The numbers are appropriately updated in the text to reflect the changes.

**Review #1**

- On the method line 79; the ethical review should be place after the study design.
  Line 83: the type of study design was not mentioned likewise sample size was determination. Thank you for this feedback. We have included the study design in lines 79-81 before the ethical review. We have added additional information about the sample size determination in lines 113-121. Citation #23 has also been added to support the sample size information for the psychometric property evaluation portion.
- On the conclusion line 230, the author stated that” the research tool can be utilized to investigate current attitude’’ I do not agree with the assertion. The instrument can only measure perception and belief. The author could remove the attitude from that statement. We have removed the word “attitude” from the sentence as suggested.

**Reviewer #2**

- The author may wish to declare the research Ethic clearance number which is usually given by the review Board. Thank you for identifying this. We have added both UCLA’s and YSMU’s IRB numbers to lines 82 and 84, respectively.
- For evidence based approach, it will not be out of context if the researchers display the English version of the items contained in the five constructs; and also the translated version which was done into Eastern Armenian Language. . A-38 item construct is huge and will be better to mention just a few if not all. We have attached both of the full surveys (English and Armenian), including the CHBMS, as supplements (Files S2 and S3)
- The authors need to subject this manuscript to grammar checks and accuracy. Couple of grammatical errors /omission of conjunctions were obvious. Thank you - we have reviewed it several times and identified a few errors that have been corrected.
- There was no clarity on how the researchers got the phone numbers of the participants. I suggest this should stated clearly on the methodology. Were there any encounters during this process of telephoning? No statement captured this. We have added additional information regarding the phone numbers and the methodology to lines 101-112. Hopefully this clarifies the methodology sufficiently.
- The authors to make available the translated version for future use by other researchers in the study area. We have added the translated version for future use as a supplement “S2”

**Reviewer #3**

- How was the random sampling done? Is it according to districts or street numbers of based on clusters within the state capital? There is need for more clarity on this statement. See 35-36 on Page 2. Within the methodology section, we have added additional information about the random sampling between lines 101-112. It is done within districts within the state capital.
- The authors should consider moving the statement on Institutional review board (IRB) on Line 79 to Line 131”. Page 4. Thank you for this suggestion. To avoid confusion between reviewers and their comments, we have kept the IRB statement in its original location.
- The authors need to provide complete details of the exemption obtained from University of California Office of Human Research Protection Program on Line 80 page 4. Complete details of exemption from UCLA have been added to line 82.
- The authors need to provide the Ethical Approval Number of the study. That is details of the ethics approval that was obtained from Yerevan State Medical University. Page 4. The Ethical Approval number from YSMU has been added to line 84.
- Why the need for stratified random approach? The authors need to provide more details. See 96 on Page 4. We have re-written this section to clarify the reasoning for stratification (please see lines 103-112).
- The Table 1 on Page 5 reports that 79(76.7%) of the respondents were uninsured and 35(34.0%) were unemployed. Based on realities in Armenian are these likely to affect the way the respondents have responded to the questions. Payment sources for mammography according to the 2016 HSPA show that approximately 39.7% of mammograms were paid for insurance or a social package (insurance: 8.3%, social package: 31.4%). It can be inferred that the remainder of mammograms were obtained without insurance or social package benefits (60.3%). Consistent with this data, 60% of Armenians pay entirely out of pocket for services not covered by the basic benefits package (e.g. mammogram). We have included a sentence to indicate that the high uninsurance rates are consistent with current Armenian realities and is likely representative of the population (line 178-181).
- The authors need to provide details and results on the 24 additional participants that are both fluent in English Language and Armenian Language. See 127 on Page 6. Additional information has been added about the English & Armenian Language participants. Please see line 129-131.
- The authors need to correct the IBM SPSS statistics version 7 to the most recent version of the software. Is this the correct SPSS version used for this study’s data analysis. See 134 on Page 6. Thank you for identifying this. It has been corrected to version “27”
